# Patients with STAT1 Gain-of-function Mutations Display Increased Apoptosis which is Reversed by the JAK Inhibitor Ruxolitinib

**DOI:** 10.1007/s10875-024-01684-y

**Published:** 2024-04-05

**Authors:** Laura Dotta, Francesca Todaro, Manuela Baronio, Mauro Giacomelli, Marinella Pinelli, Martina Giambarda, Beatrice Brognoli, Silvia Greco, Francesca Rota, Manuela Cortesi, Annarosa Soresina, Daniele Moratto, Cesare Tomasi, Rosalba Monica Ferraro, Silvia Giliani, Raffaele Badolato

**Affiliations:** 1 Department of Clinical and Experimental Sciences, Department of Pediatrics, University of Brescia, ASST Spedali Civili of Brescia, Brescia, Italy; 2 Angelo Nocivelli Institute for Molecular Medicine, Department of Molecular and Translational Medicine, University of Brescia, Brescia, Italy; 3 Department of Clinical and Experimental Sciencies, University of Brescia, Brescia, Italy; 4 Department of Pediatrics, ASST Spedali Civili of Brescia and University of Brescia, Brescia, Italy

**Keywords:** STAT1-GOF, T Lymphopenia, Ruxolitinib, T-cell Apoptosis, IGS, JAK Inhibitor

## Abstract

**Introduction:**

The signal transducer and activator of transcription (STAT1) gain-of-function (GOF) syndrome accounts for most cases of chronic mucocutaneous candidiasis but is characterized by a broader clinical phenotype that may include bacterial, viral, or invasive fungal infections, autoimmunity, autoinflammatory manifestations, vascular complications, or malignancies. The severity of lymphopenia may vary and influence the infectious morbidity.

**Methods:**

In our cohort of seven STAT1-GOF patients, we investigated the mechanisms that may determine T lymphopenia, we characterized the interferon gene signature (IGS) and analyzed the effect of ruxolitinib in reverting the immune dysregulation.

**Results:**

STAT1-GOF patients exhibited increased T lymphocyte apoptosis that was significantly augmented in both resting conditions and following stimulation with mitogens and IFNα, as evaluated by flow cytometry by Annexin V/ Propidium iodide assay. The JAK inhibitor ruxolitinib significantly reduced the IFNα-induced hyperphosphorylation of STAT1 and reverted the stimulation-induced T-cell apoptosis, in vitro. In two adult STAT1-GOF patients, the JAKinib treatment ameliorated chronic mucocutaneous candidiasis and lymphopenia. Most STAT1-GOF patients, particularly those who had autoimmunity, presented increased IGS that significantly decreased in the two patients during ruxolitinib treatment.

**Conclusion:**

In STAT1-GOF patients, T lymphocyte apoptosis is increased, and T lymphopenia may determine higher risk of severe infections. The JAKinib target therapy should be evaluated to treat severe chronic candidiasis and lymphopenia, and to downregulate the IFNs in patients with autoinflammatory or autoimmune manifestations.

**Supplementary Information:**

The online version contains supplementary material available at 10.1007/s10875-024-01684-y.

## Introduction

Heterozygous signal transducer and activator of transcription 1 (*STAT1*) gain-of-function (GOF) mutations have been discovered in 2011 [1, 2] as the cause of most inherited chronic mucocutaneous candidiasis (CMC), but follow-up studies have soon demonstrated that the STAT1-GOF disorder is extremely heterogeneous and determines an immune dysregulation syndrome characterized by a broad spectrum of bacterial, mycobacterial, viral, or other invasive fungal infections, autoimmunity or autoinflammatory manifestations, vascular aneurysms, and malignancies [3–5]. Hypothyroidism is the most frequent endocrine dysfunction, as reported in the 44% of patients described in the largest cohort study [3], albeit a wide variety of other multiorgan autoimmune diseases may complicate the phenotype, including autoimmune cytopenia, Systemic Lupus Erythematosus, inflammatory bowel diseases, or dermatological conditions such as alopecia, vitiligo, and psoriasis [3, 5, 6]. *STAT1* GOF mutations mostly affect the coiled-coin domain (CCD) or the DNA-binding domain (DBD) of the gene. The gain-of-function activity was initially ascribed to the hyperphosphorylation of STAT1 at the amino acid residue Y701 after stimulation by type I and type II interferons (IFNs) and IL-27 [2], and furtherly demonstrated through impaired STAT1 dephosphorylation in response to IFNs and IL-27, or increased STAT1 protein levels with normal pattern of dephosphorylation [7, 8]. The hyperactivity of STAT1 in response to IFNs and IL-27 signals associates with PD-L1 up-regulation, reduced expression of STAT3-dependent genes including SOCS3, and, consequently, inhibition of Th17 differentiation that leads to the susceptibility to fungal infections [9, 10]. Immunological abnormalities may vary and include T and B lymphopenia, and low IgG levels often with impaired antibody production to protein antigens. Recently, a more profound combined immunodeficiency that associates with more severe infectious, autoimmune phenotype and increased mortality has been correlated to *STAT1* mutations in the DNA-binding domain (DBD) [11]. Autoimmunity may depend upon the enhanced response to type I IFNs and a stronger IFNs-JAK-STAT1-dependent genes circuit in these patients. STAT1 signals throughout Janus kinases (JAKs) that are activated following the cytokine binding to the receptor; the JAK-mediated phosphorylation of the cytokine receptor allows the recruitment of STAT1 molecules that are phosphorylated, dimerize, and translocate to the nucleus to regulate gene expression. It follows that the inhibition of JAKs may regulate the augmented STAT1 signaling. Since 2015, the use of JAK inhibitors (JAKinib) has been reported in STAT1-GOF patients with favorable results, both with the JAK1/JAK2 inhibitors ruxolitinib (42 patients, 29 < 18 years and 4 adults) or baricitinib (6 patients, 4 < 18 years and 2 adults), or also with tofacitinib (2 children), a selective JAK3 inhibitor with limited selectivity against JAK1 and JAK2 [12–25]. Particularly, resolution or amelioration of CMC and alopecia were reported, together with the improvement of autoimmune and inflammatory manifestations. A reduction of IL-6, an increase of IL-17A and IL-17F production, and a sustained improvement of lymphopenia was demonstrated with JAKinib [13]. Conventional management includes the use of topic and systemic antifungal or antibiotic prophylaxis and therapies; variable resistance to azoles has been reported (10–39% of patients) [13], but the risk of antimicrobial resistance remains to be evaluated in follow-up studies. There is a limited experience with hematopoietic stem cell transplantation (HSCT) that was reported in 17 STAT1-GOF patients who exhibited severe disease unresponsive to conventional treatment; HSCT was burdened by secondary graft failure and death. On this aspect, the use of ruxolitinib as immunosuppressive therapy in a patient before transplant was associated to full immune reconstitution with no secondary graft loss, thus supporting the role of JAK inhibitors in controlling IFNγ-induced inflammation [16]. We have previously described nine Italian STAT-1-GOF patients who exhibited a heterogeneous phenotype whose prognosis in adolescence and adulthood depended upon the recurrence of infections, the occurrence of autoimmunity, and the development of lymphopenia. Particularly, the latter may affect long-term infectious morbidity and survival. For this reason, in the present study we aimed at investigating if T cell lymphopenia could be caused by increased apoptosis following cellular stimulation, and if the JAK inhibitor ruxolitinib could revert the pathogenesis of lymphopenia, in vitro and in vivo. Meanwhile, we chose to analyze the interferon gene signature as a biomarker of active autoimmune/autoinflammatory diseases and aimed at evaluating the effect of the JAK inhibitor ruxolitinib in modulating the IFN-driven hyper-inflammatory activation in STAT1-GOF patients.

## Methods

### Patients

The present study included seven patients who were diagnosed in our center with a STAT1-GOF mutation and previously reported [26, 27]. Informed consent was obtained from the patients or their parents if they were minors. The study was conformed to the protocols of University of Studies of Brescia and ASST Spedali Civili of Brescia and obtained the approval of the local ethical committee.

### Lymphocyte Analysis

Lymphocyte subset analyses were performed with a combination of mAbs (Becton Dickinson) according to manufacturer instructions and completed by using the FlowJo software version 8.8.7 (TreeStar). Values were compared to normal ranges established in our Laboratory from a pool of healthy subjects, according to age.

### Study of T-Cell Apoptosis by Annexin V Staining-Based Assay and Real-Time PCR

Peripheral blood mononuclear cells (PBMCs) derived from STAT1-GOF patients and healthy subjects were obtained from heparinized blood by density gradient centrifugation over Ficoll (Sigma, St. Louis, MO). PBMCs were cultured in a 24-well plate in the absence of stimulation or stimulated with coated anti-human CD3 (clone OKT3, 1 µg/mL) plus anti–human CD28 (1 µg/mL), and with the addition of IFNα (10000U/mL), or with IFNα alone. Next, ruxolitinib was added to PBMCs cultures at the dosage of 1 µg/mL. After 48-hour stimulation, cells were transferred to a 5 mL culture tube to be washed once with ice cold PBS and once with Annexin binding buffer (10 mM HEPES, 140 mM NaCl, 2.5 mM CaCl2, pH 7.4). Subsequently, PBMCs were stained with Annexin V-FITC (2 µL of Annexing V-FITC Kit, Miltenyi) and 2 µl of anti-CD3-PerCP Cy5.5 (Becton-Dickinson). Then, PBMCs were incubated for 10 min at room temperature in the dark and acquired within 1 h by FACSCalibur flow cytometer (BD Bioscence). Cells were analyzed by flow cytometry after gating CD3 + cells and assessed by the FlowJo version 7.5 Software (TreeStar). Propidium iodide (PI) was used to differentiate cells in the initial apoptotic phase (positive to annexin V, negative to PI), necrotic cells (positive to annexin V, positive to PI) and viable cells (negative to annexin V, negative to PI). We performed independent experiments with one STAT1-GOF patient and one healthy control in each at least twice and pooled the data comparing the seven STAT1-GOF patients versus healthy controls. The evaluation of CASP3 and IRF1, as markers of apoptosis, was assessed by Real-Time PCR, using the same conditions of stimulation with anti-CD3/anti-CD28 and anti-CD3/anti-CD28/IFNα, as described above, and ruxolitinib was added to PBMCs cultures at dosage of 1 µg/mL. After 48-hour stimulation, cells were pelleted at 1200 rpm for 10 min and the pellet was used to extract RNA. Quantitative reverse transcription polymerase chain reaction (qPCR) analysis was performed by the TaqMan Universal PCR Master Mix (Applied Biosystems) using CFX96 (Biorad®). TaqMan probes are: CASP3 (Hs00234387_m1), IRF1 (Hs00971965_m1). The relative abundance of each target transcript was normalized to the expression level of two housekeeping genes, ACTB (Hs01060665_m1) and GAPDH (Hs02786624_m1), as available in the experimental setting, and assessed with the CFX96 Biorad Software (BioradⓇ). Target gene expression was normalized to the housekeeping gene expression and presented as *n*-fold increase over those in the healthy control by using 2-ΔΔC T evaluation.

### Analysis of Phosphorylation of STAT1

The analysis of phosphorylation of STAT1 was conducted on peripheral blood T lymphocytes: 100 µl of whole blood were left unstimulated or stimulated with IFNα (40000U/mL for 30 min), or IFNα added with ruxolitinib, and were stained simultaneously using a fluorescein R-Phycoerythrincyanin5.1(PerCP-Cy5.5)-conjugated mouse anti-CD3 IgG mAb. Increasing doses of 0.1 µg/mL, 1 µg/mL, and 10 µg/mL of ruxolitinib were used to evaluate a dose-dependent effect. Cells were fixed and permeabilized, following the manufacturer’s instructions according to the BD protocol (Protocol III). Intracellular staining of activated STAT1 protein was performed with phycoerythrin (PE)-conjugated mouse anti-pSTAT1-Tyr-701 IgG mAb (BD Pharmigene) and isotype-matched mAb PE (BD Bioscence). CD3 + cells were gated by FACSCalibur flow cytometer (BD Bioscence) and analyzed by the FlowJo version 7.5 Software (TreeStar). The extent of STAT1 phosphorylation was calculated as Mean Fluorescence Intensity (MFI) after subtracting the MFI level of unstained cells stained with an isotype-matched mAb.

### Determination of Interferon Signature

Healthy subjects and STAT1-GOF patients derived PBMCs were re-suspended in RPMI 1640 medium, supplemented with 2 mM glutamine, 50 mg/mL penicillin, 50 mg/mL streptomycin and 10% heat-inactivated FCS (Fetal Calf Serum, Sigma, St. Louis, MO), plated in 24-multi well at a concentration of 500,000 cells/mL that were left unstimulated for 24 h. Total RNA was extracted by the RNA isolation kit NucleoSpin RNA II, according to the manufacturer’s protocol (MACHEREY-NAGEL). RNA concentration was assessed using a spectrophotometer (Infinite M200, Tecan) and analyzed by NanoQuant application. The extracted RNA was stored at -80° C until use. Retrotranscription was performing with 200ng of total RNA using the IMPROM-II Reverse Transcriptase Kit (Promega) in a total volume of 20µL, with the following steps: 25° C for 5 ‘, 42° C for 1 h and 70° C for 15 min. The cDNA obtained was stored at -20°C. Quantitative reverse transcription polymerase chain reaction (qPCR) analysis was performed by the TaqMan Universal PCR Master Mix (Applied Biosystems) using ABI PRISMTM 7000 Sequence Detection System (Applied Biosystem). TaqMan probes used are: IFI27 (Hs01086370_m1), IFI44L (Hs00199115_m1), IFIT1 (Hs00356631_g1), ISG15 (Hs00192713_m1), RSAD2 (Hs01057264_m1), and SIGLEC1 (Hs00988063_m1). The relative abundance of each target transcript was normalized to the expression level of 18S (Hs999999001_s1) and assessed with the ABI PRISMTM 7000 SDS Software (Applied Biosystems). Target gene expression was normalized to the 18S housekeeping gene expression and presented as *n*-fold increase over those of a pool of 21 healthy subjects by using 2^-ΔΔ^C T evaluation. For Interferon Score, the median fold change of the six genes of the IGS of STAT1-GOF patients was compared to the healthy control assessed as calibrator (RQ = 1).

### Statistical Analysis

The database was formatted through the Microsoft-Excel® software ver.365 and later imported from the IBM-SPSS® software ver. 27.0.1 (IBM SPSS Inc. Chicago, Illinois); the use of the Stata® software ver. 17.0 (Stata Corporation, College Station, Texas) was also considered. Normality of the distributions was assessed using the Kolmogorov-Smirnov test. Continuous variables were presented as means ± SD (in case of a normal distribution), or medians and min/max (in case of a skewed distribution) and compared with the use of the Mann-Whitney test for the analysis of the results of apoptosis tests. A *p*-value < 0.05 was considered statistically significant.

## Results

### T Lymphocyte Apoptosis is Significantly Increased in STAT1-GOF Patients

**Fig. 1:**
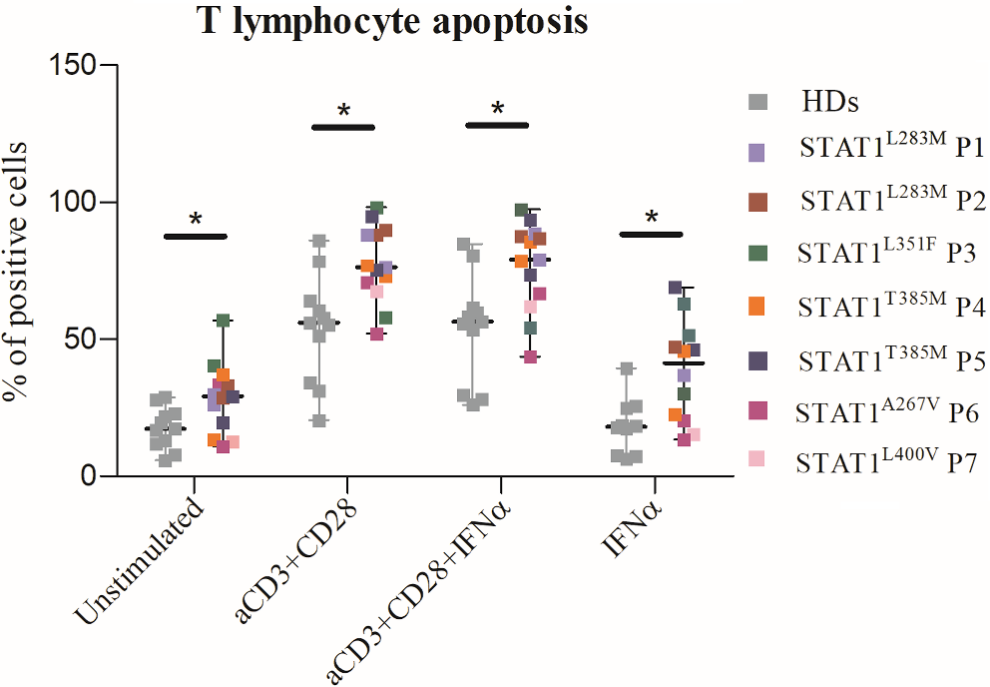
Cellular apoptosis was measured with flow cytometry in T lymphocytes that were left unstimulated or stimulated for 48 h: the results are shown as percentage of early and late apoptotic cells that were positive to Annexin V-FITC. STAT1-GOF patients exhibited significantly increased apoptosis on basal condition (unstimulated) and following all combinations of stimuli, compared to healthy subjects that were tested in parallel. Statistical significance was assessed by the Mann Withney U test with two tails, *p < 0.05

**Fig. 2:**
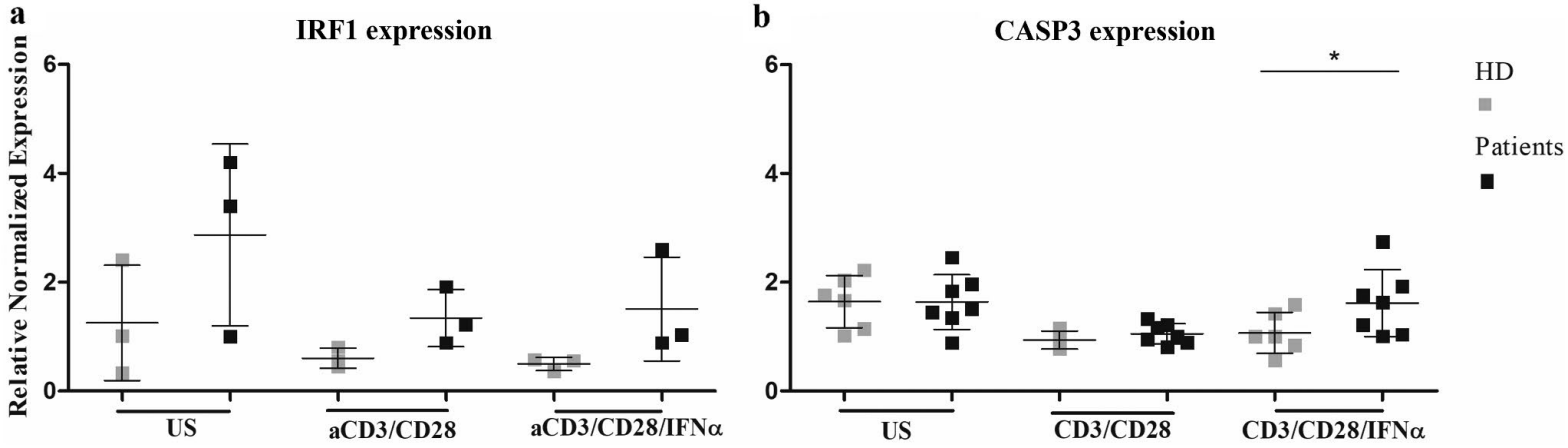
IRF1 (**a**) and CASP3 (**b**) expression was assessed by Real-Time PCR assay in healthy controls (gray color) and STAT1-GOF patients (black color). STAT1-GOF patients showed increased expression of CASP3 following stimulation with anti-CD3/anti-CD8/IFNα (*p < 0,05). *US*, unstimulated

The clinical characteristics of the seven STAT1-GOF patients who were investigated in the present study are summarized in Table 1. In our cohort, the two adult patients (STAT1^L283M^ P2 and STAT1^L351F^ P3, as named in Dotta et al. [26, 27]), had lymphopenia (absolute total lymphocyte count below 1.000 cells/mmc), while the three other adolescent/young adult patients (STAT1^T385M^ P4, STAT1^T385M^ P5 and STAT1^L400V^ P6) presented borderline counts of total lymphocytes, all with a consensual reduction of both CD4 + and CD8 + subsets. We had previously analyzed that the thymic output of all STAT1-GOF patients of our cohort was normal by evaluating the T-cell receptor excision circles (TRECs) production and the quantification of the recent thymic emigrants (RTEs) subset of peripheral T cells by flow-cytometry assay (data not shown); meanwhile, T cell proliferation in response to stimulation with mitogens (anti-CD3, anti-CD28, IFNα, IL-2) was normal in STAT1-GOF patients compared to healthy subjects (data not shown). To investigate T lymphopenia, we analyzed if STAT1-GOF T cells exhibited an abnormal pattern of apoptosis in response to stimuli. The Annexin V-FITC binding assay was used to detect apoptotic cells. Apoptosis was measured by flow cytometry in T lymphocytes that were left unstimulated or stimulated for 48 h with anti-CD3 and anti-CD28, or with anti-CD3, anti-CD28 plus IFNα, or IFNα alone, to specifically analyze the cellular response to type I IFNs. In the evaluation of the percentage of apoptotic T cells we included both early apoptosis (positive to annexin V, negative to PI T lymphocytes) and late apoptosis (positive to annexin V and PI T lymphocytes) (Fig. 1). Data showed that T cell apoptosis was significantly higher in STAT1-GOF patients compared to healthy controls in basal condition without stimulation (median of percentage of apoptotic cells 29.2 versus 17.2, respectively, *p* 0.01). 48-hour cellular stimulation induced an increase of T lymphocyte apoptosis both in healthy controls and patients, but in the STAT1-GOF patients the T cell apoptosis was significantly higher with all types of combination of stimuli (median of percentage of apoptotic cells for anti-CD3/anti-CD28 76.3 versus 56.1, *p* 0.004; anti-CD3/anti-CD28 with IFNα 79 versus 56.4, *p* 0.006; and IFNα alone 41.3 versus 18.1, *p* 0.011, respectively). Next, we evaluated the mRNA expression of the gene encoding interferon regulatory factor (IRF-1) (Fig. 2a), a transcription factor that mediates caspase activation and induction of T lymphocyte apoptosis, and of *CASP3* gene (Fig. 2b) in STAT1-GOF patients. We observed a significant increase of CASP3 expression after anti-CD3/anti-CD8/IFNα 48-hour stimulation as compared to healthy controls (*p* < 0.01). Beside IRF1 expression upregulation in STAT1-GOF patients, we failed to detect a significant difference in these experimental conditions.

**Table 1:** Summary of genotype and phenotype of the seven STAT1-GOF patients investigated in the present study

	Sex	Age (years)	Mutation (c.DNA)	Mutation (aa change)	Affected domain	CMC	Bacterial infections	Pulmonary disease	Hypothyroidism	Autoimmunity	Lymphopenia
P1	Male	19	c.(847T > A)	L283M	CCD	+/-	-	-	+	-	-
P2	Female	53	c.(847T > A)	L283M	CCD	+	+	+	-	ANA and ENA positivity, myositis	+
P3	Male	41	c.(1441G > T)	L351F	DBD	+/-	+	+	+	SLE-like skin disease, ANA and ds-DNA antibody positivity	+
P4	Male	23	c.(1542 C > T)	T385M	DBD	+	+	+	+	ANA positivity, vitiligo	+/-
P5	Male	18	c.(1542 C > T)	T385M	DBD	+	+	+	-	-	+/-
P6	Female	22	c.(801T > A)	A267V	CCD	+	+	+	-	Oral aphthosis	+/-
P7	Female	15	c.(1198 C > G)	L400V	DBD	+	+	-	-	-	-

### Ruxolitinib Reverts STAT1 Hyperphosphorylation

**Fig. 3:**
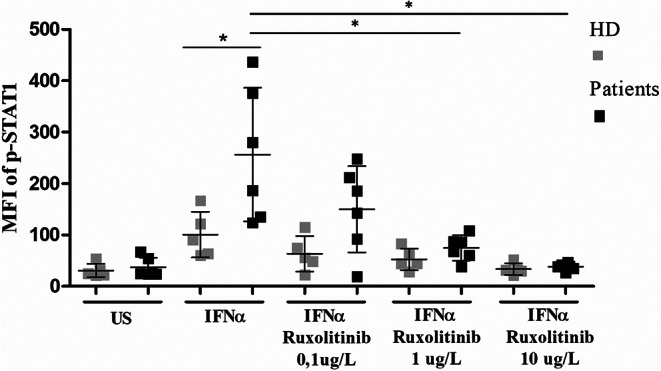
Phosphorylation of STAT1 (pSTAT1) after stimulation with IFNα was assessed by flow cytometry in STAT1-GOF patients (black color) and healthy controls (gray color). In STAT1-GOF patients the expected increase of pSTAT1 was statistically significant compared to healthy controls and was significantly reversed by the JAK inhibitor ruxolitinib with a dose-dependent effect. *US*, unstimulated

We had previously evaluated that the gain of STAT1 function in the STAT1-mutated patients of our cohort was characterized both by a trend toward an increase of pSTAT1 in unstimulated cells and a significant increase of pSTAT1 in T lymphocytes in response to IFNα (*p* 0.03), similarly to what has been reported in literature [2, 15]. Meanwhile, these findings did not associate with an increase of total STAT1. In the present study, we evaluated the efficacy of the JAK inhibitor ruxolitinib in reverting the STAT1 hyperactivation. Consistent to published data [17], we confirmed in vitro that ruxolitinib suppressed the IFNα-induced increase of STAT1 phosphorylation in T lymphocytes in a concentration-dependent manner, that was significant at the doses of 1 and 10 µg/mL, respectively (*p* 0.002) (Fig. 3).

### Ruxolitinib Decreases T Lymphocyte Apoptosis in Response to Stimulation

**Fig. 4:**
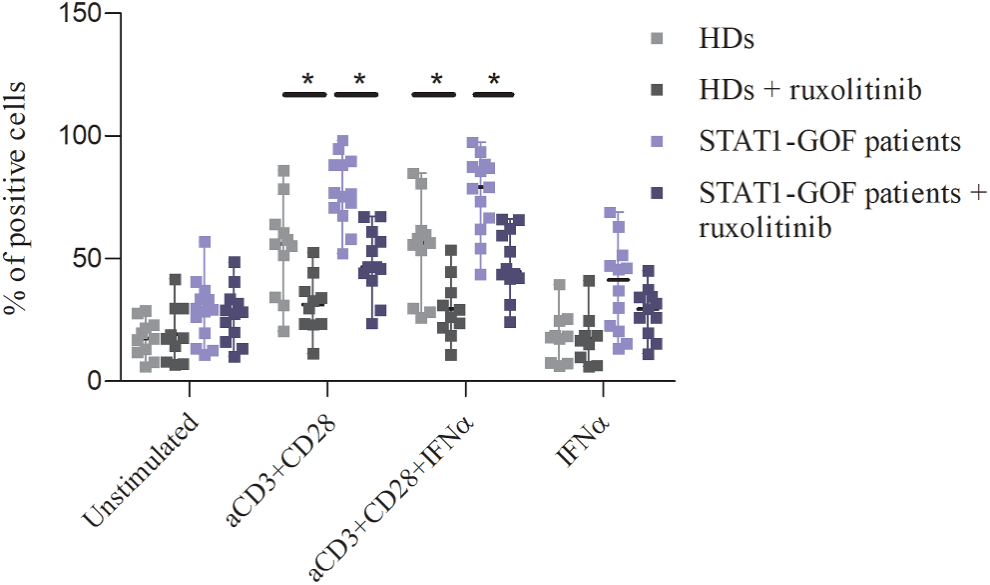
The effect of ruxolitinib on T lymphocyte apoptosis was evaluated in vitro with flow cytometry as percentage of early and late apoptotic cells that were positive to Annexin V after 48-hour stimulation: ruxolitinib significantly reduced stimulation-induced T cell apoptosis both in STAT1-GOF patients and healthy subjects (as assessed by the Mann-Whitney test)

We investigated in vitro the effect of ruxolitinib on stimulation-induced T cell apoptosis. In the same experimental condition as above described, T cell apoptosis was evaluated by flow cytometry as percentage of both early and late apoptotic cells after 48 h of stimulation with all previously indicated combination of stimuli (anti-CD3/anti-CD28, anti-CD3/anti-CD28 with IFNα, or IFNα alone), with or without the addition of ruxolitinib (1 µg/mL) (Fig. 4**)**. In STAT-1 GOF patients, ruxolitinib significantly decreased T cell apoptosis induced by stimulation by anti-CD3/anti-CD28 (median of percentage of apoptotic cells of 46.6 with ruxolitinib versus 76.3, respectively, *p* 0.0001) and anti-CD3/anti-CD28 with IFNα (median of percentage of apoptotic cells of 44.8 with ruxolitinib versus 79, respectively, *p* 0.0001). Ruxolitinib significantly reduced stimulation-induced T-cell apoptosis also in healthy controls (median of percentage of apoptotic cells following anti-CD3/anti-CD28 and anti-CD3/anti-CD28 with IFNα stimulation of 31.3 and 27.8 with ruxolitinib, respectively, *p* 0.008, versus 56.1 and 56.4, respectively, *p* 0.005).

### Ruxolitinb Treatment Response In Vivo to T Lymphopenia

**Fig. 5:**
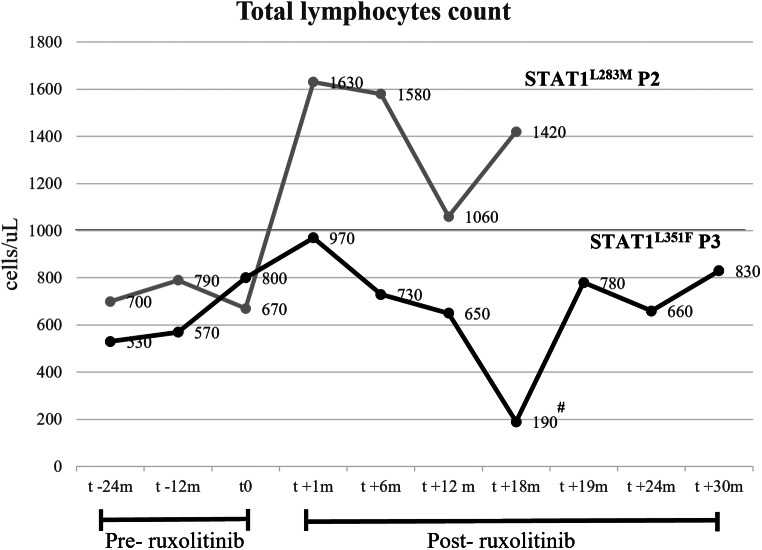
Lymphocyte count in STAT1^L283M^ P2 and STAT1^L351F^ P3 treated with ruxolitinib. #: nadir of total lymphocyte count was observed in P3 during JAKinhib treatment when he had SARS-COV2 infection and pulmonary aspergillosis

In our cohort, the two adult patients (STAT1^L283M^ P2 and STAT1^L351F^ P3) were started on the JAK inhibitor ruxolitinib. STAT1^L351F^ P3 is a 42-year-old man who suffered from a broad spectrum of infections: he manifested chronic mucocutaneous candidiasis since infancy, had esophageal candidiasis in his twenties, presented recurrent respiratory infections leading to bronchiectasis, had multiple granulomatous necrotizing lymphadenitis caused by *Cryptococcus neoformans* infection at the age of 15 years, presented recurrent molluscum and Human Papilloma Virus (warts) infections. Moreover, the patient had visceral leishmaniasis at the age of 18 years, that recurred, having the last episodes at the age of 36 and 39 years, respectively: he presented with fever, splenomegaly, and pancytopenia. He was on a regimen of itraconazole, levofloxacin and azithromycin prophylaxis, and was treated with liposomal B amphotericin when Leishmania infection recurred, with clinical response. P3 developed lymphopenia during his third decade of life, with a count of total lymphocytes of 924 ± 266 cells/mmc (media ± SD), CD4 + T cells of 339 ± 94 cells/mmc (media ± SD), CD8 + T cells of 205 ± 59 cells/mmc (media ± SD), and NK cells deficiency of17 ± 7 cells/mmc (media ± SD). Six months after the recovery of the last episode of leishmaniosis, he presented lymphopenia (570 cells/uL) and chronic thrombocytopenia (median 86.000/mmc) with mild bleeding. In P3, the main reasons to start JAK inhibition were relapsing Leishmania infection with persistent thrombocytopenia, and lymphopenia: initially, he received 5 mg orally twice daily, rapidly augmented, as well tolerated, to 10 mg twice daily. During the 34-month-follow-up time on JAKinib, we could observe only a slight increase of his total lymphocyte count, that remained over 500 cells/mmc (median 630 cells/mmc) (Fig. 5); interestingly, thrombocytopenia stably resolved (median count 260.000/mmc, data not shown), and he did not present any leishmaniosis relapse. Besides vaccination against COVID-19, the patient had severe COVID-19 manifesting with respiratory distress that required oxygen by Venturi mask and received steroids; then, he developed pulmonary aspergillosis that resolved after six-month-treatment with voriconazole. During both SARS-CoV-2 primary infection and complication,treatment with JAKinib was not suspended but reduced by a half (5 mg twice daily). While on JAKinib treatment, he had one episode of lymphadenitis. STAT1^L283M^ P2 is a 54-year-old woman who suffered from severe chronic mucocutaneous candidiasis involving oral cavity, esophagus, and genitalia since adolescence. She developed hepatotoxicity due to treatments with azoles and she did not respond to topic prophylaxis with Posaconazole. She had lymphopenia (total lymphocyte count 670 cells/mmc, CD4 + T cells 363 cells/mmc, CD8 + T cells 189 cells/mmc, NK cells 91 cells/mmc) and suffered from chronic lung disease characterized by bronchiectasis and frequent bacterial exacerbations. In P2, we decided to start ruxolitinb due to uncontrolled candidiasis and lymphopenia, with recurrent pulmonary infections. She was started with ruxolitinib 10 mg twice daily. During the 18-month-follow-up time, we observed a rapid response to ruxolitinib in term of almost complete resolution of chronic mucosal candidiasis and normalization of lymphocyte count (median 1.385 lymphocytes/mmc) (Fig. 5). P2 was fully vaccinated against COVID-19 (four doses), and experienced mild SARS-CoV2 infection with few days of fever and cough, while on JAKinib that was not discontinued during infection.

### STAT1-GOF Patients Present an Increased Expression of Interferon-Regulated Genes that Is Downregulated by Ruxolitinib

**Fig. 6:**
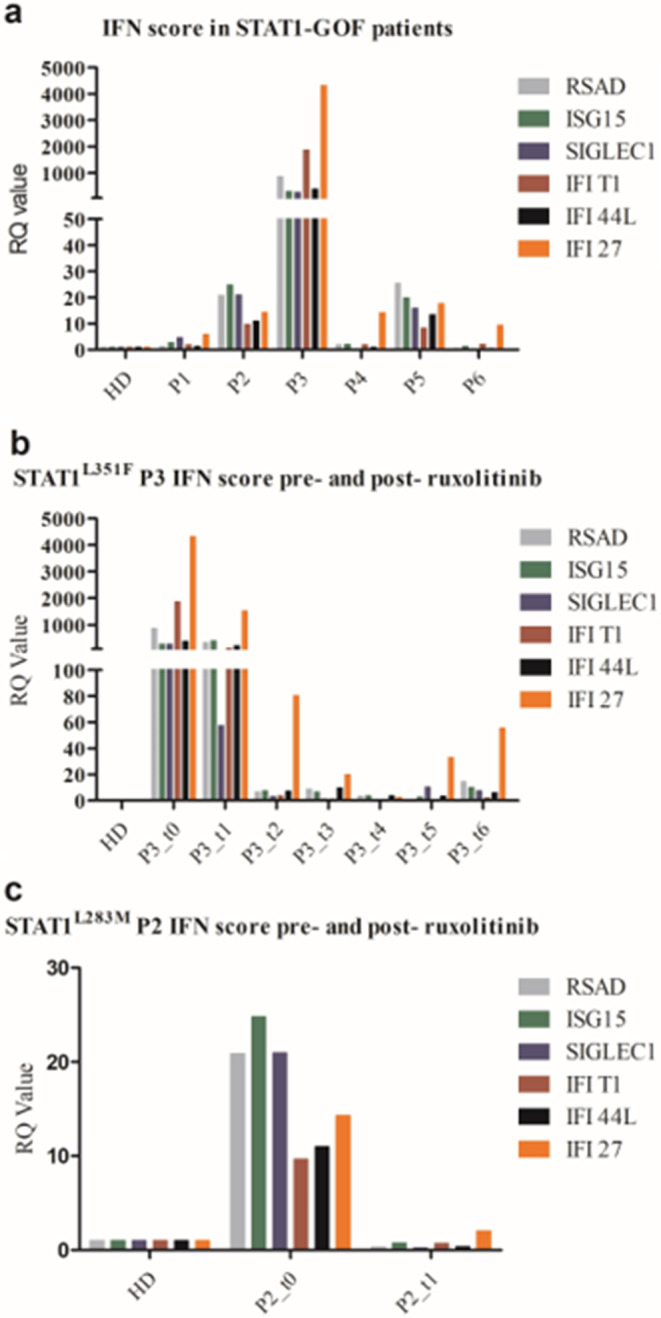
Interferon gene signature (ISG) was measured in leukocytes of STAT1-GOF patients, in comparison to a pool of healthy controls. The relative gene expression levels were calculated using the comparative Ct (∆∆Ct) method and normalized to the expression of the housekeeping gene 18S. STAT1-GOF patients presented increased ISG compared to healthy subjects (**a**), that reduced or normalized during ruxolitinib treatment in both STAT1^L351F^ P3 (b) and STAT1^L283M^ P2 (c) (in STAT1^L351F^ P3 t0 is before starting ruxolitinib, t + 1,2,3,4,5 correspond to + 1, +2, + 3, +8, + 12, and + 24 months, respectively; in STAT1^L283M^ P2 t0 is before the initiation of the JAKinib, t + 1 is after 6 months of treatment). *HD*, healthy donor

The GOF-STAT1 mutations lead to the increased expression of type 1 IFN-regulated genes in response to type 1 IFNs receptor stimulation. We herein report the analysis of the interferon gene signature (IGS) on resting leukocytes in six patients of our cohort, as available, and monitored the ISG during JAKinhib treatment. IGS was calculated using the comparative Ct (∆∆Ct) method and normalized to the expression of the housekeeping gene 18S. STAT1-GOF patients exhibited increased ISG, compared to healthy subjects, that was augmented in patients with autoimmunity (Fig. 6a). In details, STAT1^L283M^ P2 exhibited positive antinuclear antibodies (title 1:640) and extractable nuclear antigen antibodies associated with myositis, STAT1^L351F^ P3 suffered from Systemic Lupus Erythematosus skin face lesions and had a positivity for antinuclear antibodies (title 1:320) and anti-double strand DNA antibodies (138 − 122%, normal value < 35%), STAT1^T385M^ P5 had vitiligo, positivity for antinuclear antibodies (title 1:320), and presented inflammatory perianal disease, and STAT1^L400V^ P6 had recurrent complex aphthosis that responded to colchicine. Of note, STAT1^L283M^ P1 is asymptomatic for autoimmunity but the IFN score that was first negative became positive in his twenties (2.94 and 10.34, respectively). As expected, the analysis of the IFN score following IFNα stimulation furtherly showed a significant increase in STAT1^L351F^ P3, STAT1^T385M^ P4 and STAT1^L400V^ P6, compared to healthy control (data not shown). During ruxolitinib treatment, in STAT1^L351F^ P3 we observed a marked and progressive reduction of the expression of IFN genes, even though the IFN score remains positive compared to healthy controls (8.51 versus 7.24, respectively, on last follow-up, Fig. 6b). IFN score of STAT1^L283M^ P2 normalized while on treatment (19.93 versus 0.74, respectively), compared to healthy controls (IFN score 7.24) (Fig. 6c).

## Discussion

Patients with STAT1-GOF mutations exhibit a broad spectrum of infectious, inflammatory, vascular, and neoplastic manifestations. The conventional treatments include antimicrobial prophylaxis, appropriate treatment of acute or chronic infections, and immunosuppressive drugs for autoimmune/autoinflammatory manifestations. Hematopoietic stem cell transplantation may represent a curative strategy in patients with severe combined immunodeficiency or non-responders to various immunosuppressive agents, but the prognosis remains poor also considering the elevated morbidity when the transplant had been considered after infancy [14, 15]. As for most inborn errors of immunity, the younger age at transplant seems the strongest positive indicator of overall and event free survival. JAK inhibitors (i.e. ruxolitinib) may represent a target therapy in STAT1-GOF patients. Currently, its use is promising in terms of treating severe forms of candidiasis, alopecia areata, autoimmune/autoinflammatory phenomena, and in controlling IFNs-induced inflammation in the setting of transplantation, in order to improve engraftment rate and overall outcome [14–25]. However, some authors have reported the therapeutic failure of ruxolitinib in treating severe fungal infections other than CMC, or early-onset inflammatory bowel disease [19, 24, 25], suggesting that larger cohort studies are recommended to support the efficacy of JAK inhibitors in STAT1-GOF patients. In the STAT1-GOF syndrome, lymphopenia has been variably reported [3, 5, 27–31]. Previous studies have investigated the role of STAT1 in cell growth and apoptosis [32,33,34]: STAT1-negative U3A fibroblast line underwent growth arrest upon transfection with wild type STAT1α and treatment with interferon-γ [34], and prolonged nuclear localization of activated STAT1 resulted in apoptosis involving specific regulation of caspase pathway. Romberg et al. [35] reported progressive B-cell lymphopenia in two patients carrying the E235A allele, that was associated with increased Annexin V staining of CD19 + B cells after 24 h of culture and elevated caspase activity. In the present study, we investigated T cell apoptosis in STAT1-GOF patients and the risk of lymphopenia in these patients, despite normal human thymic output. We observed that, compared to healthy subjects, T lymphocytes from all analyzed STAT1-GOF patients presented increased T cell apoptosis both in unstimulated condition and after 48-hour stimulation with mitogens, particularly augmented with the combination of signals via CD3, CD28 and IFNα pathways. Regarding T cells, there is evidence that either anti-TCR or anti-CD3 can enhance or maintain lymphocyte viability, or in some circumstances induce apoptosis. STAT1 hyperfunction in mitogen-activated T cells might accelerate the pathway of programmed cell death via caspase activation in response to IFNs stimulation. Our observation of an increased expression of CASP3 in STAT1-GOF patients was limited to the condition of CD3, CD28 and IFNα stimulation, with a trend toward an increase of IRF expression but without any significant differences in the low number of analyzed patients and our experimental settings. The apoptotic signaling should be furtherly investigated in larger cohort of patients to characterize the role of the gain-of-function of STAT1 in regulating the transcription and expression of apoptotic markers and the effect of JAKinib in regulating apoptosis in STAT1-GOF patients. Consistent with previous reports, we confirmed that treatment of IFNα-cultured T cells with the JAK inhibitor ruxolitinib significantly reversed the hyperphosphorylation of STAT1 in a dose-dependent manner. Moreover, we observed that ruxolitinib reduced T cell apoptosis that was induced by cellular stimulation in vitro; in the unstimulated condition, ruxolitinib variably reduced T cell apoptosis but without any significant difference. The precise mechanism of ruxolitinib in reverting apoptosis in vitro remains uncertain, thus explaining why the effect in vivo may be variable but influenced by the modulation of other signaling pathways. in vivo, STAT1^L351F^ P3 benefited from the JAKinib treatment in terms of control of relapsing visceral leishmaniosis and stably normalized platelet count; the total lymphocyte count did not normalize but remained stable above 500 cells/mms. However, the time of follow-up is too short to get prove of efficacy of ruxolitinib regarding reduced risk of infections, including visceral leishmaniosis. STAT1^L283M^ P2 showed normalization of total lymphocytes count (above 1000 cells/mmc) and improvement of mucocutaneous candidiasis after initiation of ruxolitinib. In patients with STAT1-GOF mutations, lymphopenia represents a negative prognostic factor because it may cause higher risk of infections, also sustained by opportunistic pathogens [3, 13]. Meanwhile, we may hypothesize that the recurrence of infections may contribute to the development of lymphopenia following stimulation-induced T cell apoptosis. Although we could not identify a specific mechanism linking lymphopenia to apoptosis, our results may suggest that the use of a JAK/STAT inhibitor may control the signaling unbalance in the STAT1-GOF disorder and revert the increased T cell apoptosis. The evidence that STAT1-GOF patients exhibit increased phosphorylation of STAT1 and increased T cell apoptosis even in the unstimulated state suggests that in certain circumstances patients with GOF-STAT1 syndrome display overlapping features with type I interferonopathies. This group of inborn errors of immunity includes genetic conditions associated with hyper-regulation of type I interferon [36]. In patients with interferonopathies, the analyses of mRNA expression of genes regulated by type I interferon, which is also known as IFN score, constitutes a specific marker of disease activity. In our study, we have shown that STAT1-GOF patients can exhibit increase of mRNA expression of the same subset of genes which are upregulated in patients with interferonopathies even in unstimulated cells. Interesting, we have observed that the IFN score was higher in the STAT1-GOF patients with autoimmune manifestations, albeit a correlation between the IFN score and the occurrence of clinical manifestations remains uncertain. The IFN score decreased from the first months after initiating the JAK inhibitor in our two treated STAT1-GOF patients. We propose that the analysis of IFN score in STAT1-GOF patients might be a useful marker to identify those patients with an increased risk of developing autoimmune complications. In these STAT1-GOF patients, treatment with JAKinib should be considered as a target therapy that may modulate the inflammatory response. Within our cohort, P3 experienced COVID-19 pneumonia: despite completing the vaccination cycle, he had B lymphopenia and lacked S1-specific antibody levels; in his case, the steroid treatment during SARS-CoV2 infection could have contributed to pulmonary aspergillosis. P2 experienced mild SARS-CoV2 infection without secondary complications. Recently, fatal COVID-19 infection has been reported in two children with STAT1-GOF variants due to overwhelming deregulated immune response [37]. In our STAT1-GOF patients, we can hypothesize thatthe concomitant treatment with a reduced dose of JAKinib during SARS-CoV2 infection might have balanced the risk of hyperinflammatory complications after COVID-19. A major limitation of the present study is the small sample size that may affect the significance of the results that should be confirmed in larger cohorts.

## Conclusions

The outcome of patients with STAT1-GOF syndrome may depend on the great heterogeneity of clinical and immunological manifestations, and the further characterization of the pathogenetic mechanisms is warranted to address target treatments. JAK inhibitors may be effective in controlling chronic mucocutaneous candidiasis, the hyperactivation of the interferon response, that contributes to autoimmunity and autoinflammation, lymphopenia and severe viral and opportunistic infections in STAT1-GOF patients.

### Electronic Supplementary Material

Below is the link to the electronic supplementary material.

**Supplementary Fig. 1** Dot-plot analysis of T lymphocyte apoptosis following stimulation with anti-CD3/anti-CD8, anti-CD3/anti-CD8/IFNα, or IFNα alone, and the addiction of ruxolitinib, in a patient and a healthy control, as a representative experiment of Annexin V assay

## Data Availability

No datasets were generated or analysed during the current study.
