# Supplementary figures and images for: Patients with STAT1 Gain-of-function Mutations Display Increased Apoptosis which is Reversed by the JAK Inhibitor Ruxolitinib
Source: J Clin Immunol. 2024 Apr 5;44(4):85. doi: 10.1007/s10875-024-01684-y (PMC10997685; doi:10.1007/s10875-024-01684-y)

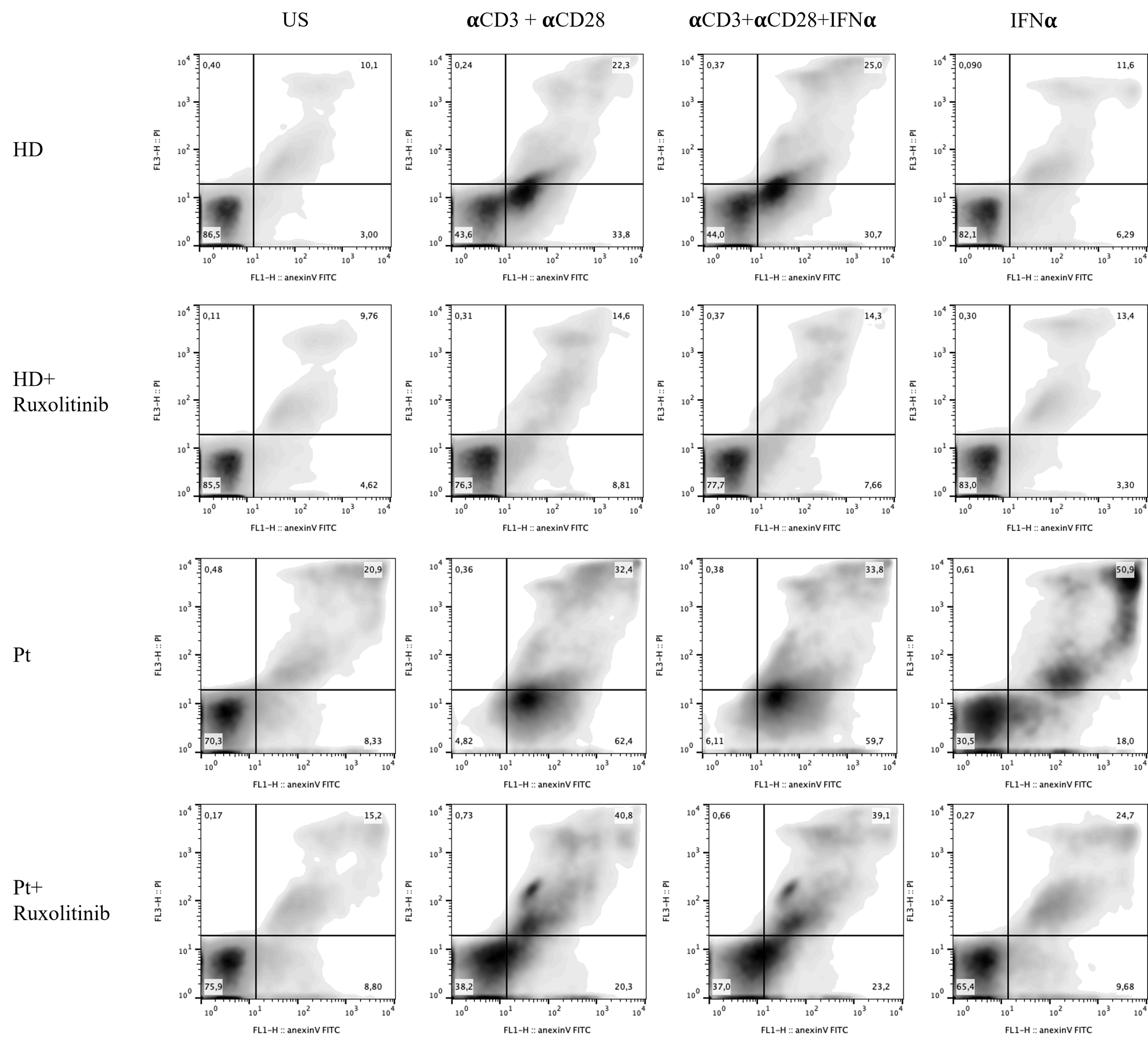

Supplement: Supplementary file 1 — Supplementary Fig. 1 Dot-plot analysis of T lymphocyte apoptosis following stimulation with anti-CD3/anti-CD8, anti-CD3/anti-CD8/IFNα, or IFNα alone, and the addiction of ruxolitinib, in a patient and a healthy control, as a representative experiment of Annexin V assay [file 10875_2024_1684_MOESM1_ESM.tif]
